# The Problem of Medical Education

**Published:** 1912-12

**Authors:** Walter C. Swayne

**Affiliations:** Obstetric Physician, Bristol Royal Infirmary, and Professor of Obstetrics, University of Bristol


### THE PROBLEM OF MEDICAL EDUCATION.

The Presidential Address, delivered on October 9th, 1912, at the opening of the Thirty-ninth Session of the Bristol Medico-Chirurgical Society.

BY

WALTER C. SWAYNE, M.D. Lond. and Bristol,

*Obstetric Physician, Bristol Royal Infirmary, and Professor of Obstetrics, University of Bristol.*

---

It has been the rule that the President of this Society should commence his year of office by delivering to the members an address, which has usually dealt with some subject of a scientific or medical nature.

In addressing you on "Some Problems of Medical Education" it may appear that the subject is somewhat outside the purview of a Society such as this, whose chief object is the discussion of medical matters from their practical or scientific aspect.

I may, however, give as one reason that the Society is meeting in the buildings of the University of Bristol, of which many of the members of the Society are also members. Another reason lies in the historical fact that in England, at any rate, medical education has been carried on by the practitioners of medicine themselves without State aid, and with only such modest pecuniary endowments as the profession itself could supply.

The corporations (other than Universities) which deal with the licensing of medical practitioners, originated as Guilds, which in their inception were more concerned with the disciplinary regulation of practice than with either teaching or licensing. They undertook, as a later development, the duty of testing the knowledge of those who proposed to practise medicine. None of the English corporations have ever engaged in the teaching of students, although the lectures given at the London Colleges might seem to suggest that they have done so

These lectures are really postgraduate in character. The Irish and Scottish Colleges of Surgeons, however, both teach and examine students.

The teaching of English medical students was carried out originally by apprenticeship to individual practitioners. Medical schools, except in the older Universities, arose almost invariably owing to the desire of those practitioners who happened to form the staff of a hospital, for the better instruction of their pupils in the scientific or academic side of the subject. Hence the close connection between the London hospitals and their schools, which have always formed an integral part of the former as far as the staff was concerned.

In London again, as also in Dublin, there flourished quite a respectable number of proprietary schools (such as Grainger's and Cooke's, the latter still exists) which had no connection whatever with hospitals, and were entirely the private ventures of individuals. In the provinces all the medical schools have become the medical faculties of modern Universities, although their origin was as a rule the same as that of the London schools.

Until quite recent years neither the medical faculties of the older Universities nor the medical schools, other than those closely connected with hospitals, ever exercised any control over clinical teaching, the existence and organisation of which is due to the fact that the honorary staff of the voluntary hospitals almost invariably obtained the privilege of taking their apprentices with them to see the practice of the hospital.

Even as late as my own student days some curious customs were preserved in the older hospitals in London which owed their origin to this. At Guy's in my time no one except a surgeon's dresser would be appointed house surgeon. In the times of my immediate predecessors (uncle and grandfather) no one except a surgeon's apprentice could become a dresser.

It is quite clear that with very few exceptions all the medical schools in Great Britain were originally proprietary. They concerned themselves entirely with the scientific teaching and practice of the preliminary and intermediate stages, and the academic side of the final stage of the medical curriculum.

Except in those London schools which were attached to hospitals, there was no control of, or any connection between, the teaching of clinical medicine and that of the academic and scientific sides of the subject. This control, however, does exist in some of the Scottish Universities, and in the University of Dublin (owing to Sir Patrick Dun), and is coming into existence to some extent in some of the more modern Universities.

The proprietary character of English medical schools, whose teachers were paid simply by fees received from pupils, led, as the expense of the scientific side of medical teaching progressively increased, to the amount of fees received by the teachers being reduced to a minimum. It was soon found that in order that adequate instruction might be provided in the preliminary and intermediate sciences, it was essential that these should be taught by scientific experts, who could give time to the supervision of laboratories which a medical practitioner could not do.

The expense of laboratory accommodation for the teaching of the preliminary and intermediate sciences also became progressively greater.

The pecuniary difficulty is not by any means the most serious side of the matter. The question of the emoluments of the teachers is a comparatively small one in comparison with the effects which have necessarily been produced on medical education as a whole. As it gradually became evident that the expense of providing training (as opposed to teaching) in the preliminary sciences, more particularly in Physiology, was increasing, one medical school after another either handed over its medical faculty to the local University or University College, or ceased to teach in many cases the preliminary, and in some cases even the intermediate sciences.

Now the effect of this on the instruction of students in these sciences is by no means to be deplored. The training provided either in the science faculties of the Universities or in the surviving medical schools, strengthened as they are by the reception of students transferred from those which have ceased to teach these subjects themselves, is far superior to that which can be afforded by a proprietary school under its original conditions and pecuniary disabilities. But this is the very thing which has led to some of the problems with reference to which I am addressing you.

The divorce between the preliminary and intermediate sciences and the study of the most important subject of the final stage, viz. Clinical Medicine, has been accentuated by this process of absorption and amalgamation. One problem which we have to face is the extent to which it is possible, without increasing the length of the curriculum, and thus adding to its already heavy expense, to adjust the time allotted to the different stages, so that while adequate attention can be given to the preliminary and intermediate sciences, sufficient time is left for clinical instruction and the study of Pathology and Bacteriology. One method by which this may be effected is by ceasing to provide teaching in the purely academic side of the final subjects, and thus allow more time to be devoted to clinical work and observation. This is being effected to a certain extent by the gradual diminution of the number of lectures the student is compelled to attend in each course.

The second alternative is to reduce to some extent the scope and intensity of the teaching in the sciences. It appears to be extremely difficult to do this by shortening the period allotted to them, and giving the time so saved to clinical instruction. Something, however, may be done by eliminating, to a certain extent, the system of water-tight compartments with its clear division between different stages, and introducing a certain amount of clinical study into, at any rate, the second and third years of the curriculum as at present arranged. This might easily be done if we could induce the teachers of the aforesaid scientific subjects to lay down courses which would not take up the whole time of the student. We cannot, however, hope for much from a move in this direction. It appears to be quite impossible to give that grounding in the laboratory work of these sciences, which is essential for the study of the more advanced subjects, if the time allotted to them is curtailed. If the student is to have a correct understanding of the chemical processes and manipulative work involved in Physiology and Pathology, the proper use of the complicated instruments, and the carrying out of the complicated investigations required in the modern practice of clinical medicine and surgery, thorough laboratory training is absolutely necessary.

There is yet another possible solution to this problem, which is to carry the commencement of specialisation farther back and into the schools, the scientific basis on which the study and practice of medicine rest being laid before the student enters the medical school or the University. We are here faced with the same difficulty which has been already alluded to in the original proprietary position of the medical schools. Practically all the schools which provide a type of secondary education suitable for students of medicine are proprietary institutions, and unable to meet the expense of providing the necessary laboratories and teachers, even when endowed. Moreover there is the difficulty of inducing their governing bodies to see that there is any necessity for them to provide the necessary but expensive laboratories and experimental apparatus for the complete instruction of their scholars in these sciences. This is not altogether surprising when we consider that it is only comparatively recently that the Universities have begun to rise out of the rut of mid-Victorian ideas of instruction in science, which consisted mainly in giving set lectures illustrated with experiments, and in the endeavour, I quote Sir William Ramsay, " to teach pupils to know something instead of to do something."

If the schools made the same provision for the teaching of science as they do for the teaching of humanistic subjects there would be no difficulty whatever. In most High Schools the teaching of humanistic subjects is far above the standard of the freshman at a University, and as far as the humanities are concerned the connection between the school and the University is almost ideal. There is no valid reason that I can see why the same connection should not exist in science.

I have just sketched one difficulty which calls for removal if possible, and have given you a brief indication of the methods by which this might be effected, but unfortunately the remedy is not in our hands.

The education of the medical student is complicated by another factor. Medicine is a profession which is partly technical and partly scientific. The present tendency is towards the continuous and relatively more rapid advance of the scientific side as compared with the technical, but let us not forget that the technical side also advances as experience accumulates. So far, however, the distribution of the periods allotted to different parts of the curriculum does not recognise that the time allotted to the technical side remains nominally the same as it was thirty years ago, and is actually reduced by the requirements of modern pathology. How this difficulty is to be met in the future it is impossible to see, since the demands of the scientific side will certainly not diminish, but rather increase.

Now follows another problem, which is to a certain extent political in nature. The advent of the Insurance Act and the introduction of medical benefits under it will inevitably lead to the separation of those following the profession of medicine into two grades—those who are to do contract practice, and the physicians, surgeons and specialists who do not. This separation, I may remind you, obtained up to the days of our grandfathers, one grade being that of the apothecary, and the other that of the physician.

It is obvious that the same training, although theoretically desirable for both grades, will become impossible as the scientific side of medicine increases in its extent and consequent demands on the student. The profession of medicine, on account of the expense involved on entering it, will be closed to those who would be satisfied with the rather exiguous income likely to be obtained under the contract system. The inevitable result will be, if this difficulty is to be met, that Government will have to intervene and provide funds which will allow of the entry to the profession of medicine of a large number of those who are at present precluded from doing so by the expense involved.

It is almost inconceivable that Government will do this on any such generous basis as will allow of the increasing demands of the scientific side of medicine receiving adequate attention, and the next step will be the elaboration of a less exacting and shorter curriculum to suit the future contract practitioners.

Another question arises here with regard to the later stages of the curriculum which may become very difficult in the not distant future, and in view of changes which may be produced as the result of recent political action.

Anything which tends to produce material alteration in the conditions under which hospital work is carried on may have, as a side issue, quite unforeseen results on medical education.

For example, the administration of maternity benefit under the Insurance Act, as at present worded, actually penalises the woman who is confined in a lying-in hospital, or attended as a patient of an out-door maternity charity. If this has the effect one would expect, we may look for an enormous rise in puerperal mortality in the future, first, because of the difficulties of instruction owing to lack of material, and, secondly, because while in lying-in hospitals the average septic mortality is 0.23 per cent. (Arnold Lea), the average septic mortality in England and Wales is 2.7 per cent., more than ten times as great. The patients will probably prefer the greater risk of being confined at home and receiving a cash bonus of 30s., although attended by a mortality of 2.7 per cent., to entering a hospital and sacrificing the cash, but with a risk of only 0.23 per cent.

Should the usefulness of general hospitals be impaired by financial loss, and their occupants reduced in number, the education of the medical student will suffer, and the population in the near future will have less efficient medical attendance. It may here be pointed out that the business of the English hospital, supported as it is by charity, is entirely the cure or relief of the sick poor, and not in addition, as in the State-supported hospitals of the continent, the provision of means for the instruction of those studying medicine. That our hospitals are great teaching centres is entirely due to the old system of apprenticeship, and to the fact that the original members of their staffs, in practically all cases, agreed with the lay managers that in return for giving their services they should have the right of taking their apprentices to see the practice of the hospital, and to assist them in carrying it on.

It is to the old system of apprenticeship that English methods of clinical teaching (of which Flexner says they as nearly approach the ideal as is possible) are due. The close association between the teachers and their pupils, the common *esprit de corps* and *camaraderie* in what, in the large hospitals, is even called the " Firm," is an undoubted survival of the old close association between master and apprentice.

Whatever changes may be in store, let us hope that this relationship will survive, but I fear that should the continental system of State-provided hospitals be introduced into this country, judging by the results of State management of other educational institutions, we may expect the teaching of medicine to be swaddled in the bonds of red tape, and governed by a syllabus imposed by those who are either ignorant or whose knowledge is out of date.

The relation of Universities to medical teaching is another matter which presents a large field for speculation.

What should be the relation of a University to medical education ?

As regards the purely scientific and academic side of medical teaching there is no difficulty, since in all modern Universities this side is adequately provided for, in fact far better so than could be done by any proprietary school of medicine unattached to a University. When we come to consider further the relation between the University and medical teaching in the final stage of the curriculum, the position is by no means ideal. As things stand the University has no control whatever over the final part of the curriculum except in Pathology, and only in this case does the proper relation obtain if the University Pathologist has in addition an appointment as pathologist in a local hospital. Any arrangement by which the University Pathologist is excluded from the pathological department of the local hospital is unsatisfactory in every way, as he becomes dependent for material on the goodwill of the hospital pathologist.

As regards the remaining subjects of the final part, viz. Clinical Medicine, Surgery, Obstetrics, etc., there is at present no definite connection between the University and the hospitals where this teaching is given. This, from an educational point of view, is an undesirable condition of things. Universities should be able to govern to some extent the teaching in clinical subjects of those students who are candidates for their own degrees. This at present they are unable to do, except as to the amount of clinical teaching which they can insist upon the condidates receiving before they can be allowed to enter for their final examination.

The ideal connection by which the University can govern to some extent the actual teaching is a very difficult thing to bring about, for one reason because the Universities as such have no control, and cannot have any control over the management of the charitable institutions in which alone this teaching can be given. Another reason is because the conditions under which students, University or otherwise, work in these institutions are based on the old apprenticeship system. It might be possible to arrange, by mutual consent, a scheme by which a certain amount of control should be given to the University over the teaching given in the local charities.

There is another point which should be considered here. To what extent should the University undertake instruction which is purely technical ?

A large part of the training of the medical student in the final stage of his curriculum is technical in essence, and it may be said that to give technical training pure and simple is not the business of the University. This, I think we must admit, is true to a very large extent, and this form of training, therefore, should remain in the hands of those who at present give it. It should be remembered, however, that in what may be called the purely technical side of medical teaching there arise certain well-defined scientific general principles, and in giving instruction in these principles the University may certainly claim to be performing its true function.

The organisation of postgraduate instruction and research is unquestionably the proper function of the University, and with regard to this the University may advance a just claim.

As a concrete illustration of the idea I am endeavouring to put before you, I would make this suggestion, that the University should appoint, with the consent of the committees and staffs of the local hospital, teachers who should be responsible to it for the instruction of the students in the earliest stages of their clinical work, in order that they may receive a thorough grounding in the principles of Clinical Medicine, Surgery, etc. This stage completed, the student should be allowed to pass on to other teachers, not necessarily appointed by the University, for the purpose of obtaining practice in the application of clinical methods, the general principles of which he has already acquired at the hands of teachers appointed by the University.

In order that such a scheme might be carried out, it would be necessary that a certain number of the beds available in the hospitals should be allocated to teachers appointed by the University for the purpose of instruction. The number need not be large, an average of three or four beds per student in any given subject is quite sufficient, as the instruction given and the work demanded of the student should be so thorough as to practically engage the whole of his working day.

After the completion of this period he should be capable of dealing with a far larger number of patients accurately and systematically.

I should like to express my personal opinion, which you must take for what it is worth, that the ideal system lies in a thorough elementary grounding given by teachers appointed and paid by the Universities, followed by the more general lines of clinical practice at present obtaining, which having a systematic basis might be much extended without harm being done by over-diffusion.

The postgraduate teaching should be, for the most part, the care of the University, and research work should most certainly be organised, carried on and paid for by the University.

In my opinion the teaching of clinical medicine is a subject no less worthy of the attention of the University than is the teaching of Practical Chemistry, Physics and Zoology, the hospital taking the place of the laboratories of the three subjects mentioned.

Unfortunately, however, the enormous expense involved in maintaining hospitals prevents their provision for the purpose of acting as the laboratories of the departments of medicine of Universities.

You may, perhaps, be interested to hear that Sir Patrick Dun's bequest to the University of Dublin was for this very purpose, but was found quite inadequate to provide sufficient clinical material to make the scheme a success. Trinity College, however, still has certain important rights over Sir Patrick Dun's Hospital in Dublin, which practically give the University the control of a certain number of beds in the hospital.

As to the duties of the University in the organisation of postgraduate instruction there can, I think, be little doubt.

As regards Research in Clinical Medicine, the fact of the matter is that in a very large number of institutions research work cannot be carried on without the co-operation of University establishments on account of the expensive equipment necessary, and the further frequent, though not invariable, necessity of making use of experts in Biology, Chemistry, Physiology and Physics, and other purely scientific subjects.

As a concrete instance, I may say that I have lately found it necessary to make use of the organic chemist, the zoologist and the physiologist of the University for the purpose of checking and correcting certain investigations which were being carried on in my department at the Infirmary.

The co-ordination of the University and the charitable institution as regards research work is a necessity in order to avoid duplication of expense and labour, and to secure the advantage that where medical research enters the domain of a pure science, it can be dealt with by experts in that science.

Should the University provide a medical course for other than those who are candidates for its own degrees? On theoretical grounds one would be inclined to say, "No, the University should concern itself with the education of its own graduands only." Practically, however, this attitude cannot be maintained, since the geographical distribution of schools of medicine is such that a large number of students would be absolutely excluded if the Universities adopted this attitude. The extent, however, to which a University school should cater or advertise for students who do not intend to take its degrees, and consequently do not intend to follow its curriculum whole-heartedly, should be very limited, and that such a course should be pursued at all is, in the opinion of many, very questionable.

In laying before you the different problems which I have touched upon in this address, I hope you will credit me with having endeavoured to avoid dogmatism. Any opinion which I have expressed as my own in the course of it, has been propounded more as a basis for argument than as a final expression. I hope that you will, each and all, give your advice and assistance to those of us on whom will fall the duty of endeavouring to co-ordinate the conflicting demands which the various subjects of medical education make upon the time of the student.

